# Complete genome sequences of *Edwardsiella ictaluri* strains isolated from ayu (*Plecoglossus altivelis altivelis*) in the Kagami and Shimanto Rivers, Kochi, Japan

**DOI:** 10.1128/mra.00435-24

**Published:** 2024-06-25

**Authors:** Masayuki Imajoh, Akane Yukawa, Miyu Kawahara, Masanori Daibata

**Affiliations:** 1 Faculty of Agriculture and Marine Science, Graduate School of Integrated Arts and Science, Kochi University, Kochi, Japan; 2 Department of Microbiology and Infection, Kochi Medical School, Kochi University, Kochi, Japan; DOE Joint Genome Institute, Berkeley, California, USA

**Keywords:** *Edwardsiella ictaluri*, ayu

## Abstract

The complete genome sequences of seven *Edwardsiella ictaluri* strains, isolated from the kidneys of dead ayu (*Plecoglossus altivelis altivelis*) in Kochi’s Kagami and Shimanto Rivers, Japan, were determined. Multilocus sequencing typing revealed that their genotypes were sequence-type ST26.

## ANNOUNCEMENT

The genus *Edwardsiella* belongs to the *Hafniaceae* family and has five valid species (1). *Edwardsiella ictaluri* causes several diseases in various fish species, including enteric septicemia in channel catfish, bacillary necrosis in striped catfish, redhead disease in yellow catfish, and edwardsiellosis in tilapia (2). Ayu (*Plecoglossus altivelis altivelis*) is a popular target fish for fisheries and anglers, particularly using the tomozuri fishing method in Japanese rivers. Since 2007, *E. ictaluri* infections have led to substantial mortality among ayu populations during the summer months (3, 4). The Kagami River, spanning 31 km, and the Shimanto River, with a 196 km length, are located in Kochi Prefecture on Shikoku Island, Japan. Freshly dead ayu fish, caused by *E. ictaluri*, was collected from the two rivers. Subsequently, seven strains were isolated from the kidneys of these dead fish as per the manual by the Japan Fisheries Research and Education Agency (https://nria.fra.affrc.go.jp/sindan/kenkyu/pdf/e_ictaluri_byougyo.pdf). Herein, the complete genome sequences of the isolated strains, namely KH20906, KB20921, KH201010, STU22726, STU22816, STA22820, and STH22820, are presented.

Frozen stocks of the seven strains were cultured on brain-heart infusion (BHI) agar plates (BD Difco, USA) at 25°C overnight. A single colony was selected, inoculated into 10 mL of BHI broth, and incubated with shaking at 25°C overnight. Following high-speed centrifugation, genomic DNA was extracted from bacterial pellets using the Wizard HMW DNA Extraction Kit (Promega, USA). The purified DNA underwent both Illumina and Nanopore sequencing. For Illumina sequencing, genomic DNA was fragmented and processed to achieve an average size of 330 bp using the QIAseq FX DNA Library Kit (QIAGEN, USA). Following adaptor ligation, 20 pM pooled libraries were sequenced on an Illumina MiSeq (MiSeq Reagent Kit v3; 150 cycles, USA), generating 75 bp paired-end reads. Miseq raw reads with Phred-encoded quality scores above 30 were selected for assembly using Trim Galore v.0.6.5 (5). For Nanopore sequencing, genomic DNA underwent treatment with the 1D Ligation Sequencing Kit (SQK-LSK109, Oxford Nanopore Technologies, USA) with Native Barcoding Expansion (EXP-NBD104). After adaptor ligation, the pooled libraries were sequenced on MinION with an R9.4.1 flow cell controlled via MinKNOW software v22.05.5. Base calling was performed using Guppy v.4.2.3 in the accurate mode implemented in MinION software. MinION raw reads below a minimum quality score of 8 were discarded using NanoFilt v.2.8.0 (6). A hybrid assembly of Miseq short reads and MinION long reads was achieved using Unicycler v.0.4.8 (7). Genome error correction, circularization, and rotation were implemented in the Unicycler pipeline. Results indicated that seven strains possessed one chromosome and two plasmids. Gene annotation, taxonomy verification, and genotyping were performed using the DDBJ Fast Annotation and Submission Tool v. 1.2.0 (8) and the multilocus sequencing typing (MLST) databases for *Edwardsiella* spp. (9). Complete genome characteristics are shown in Table 1. Notably, MLST analysis revealed that all seven strains shared sequence type ST26, similar to strain 2083 from striped catfish in Thailand (10) and strain 669 from yellow catfish in China (11).

**TABLE 1:** Characteristics of the complete genome sequences of seven *E. ictaluri* strains

Collection location(Latitude, longitude)	Kagami river (33°37'28.9"N, 133°29'43.0"E)	Kagami river (33°37'27.0"N, 133°29'09.9"E)	Kagami river (33°37'28.9"N, 133°29'43.0"E)	Shimanto river (33°11'18.5"N, 132°58'17.6"E)	Shimanto river (33°11'18.5"N, 132°58'17.6"E)	Shimanto river (33°11'19.9"N, 133°02'52.1"E)	Shimanto river (33°09'58.1"N, 133°03'35.9"E)
Collection date	September 9, 2020	September 21, 2020	10 October 2020	July 26, 2022	August 16, 2022	August 20, 2022	August 20, 2022
Strains	KH20906	KB20921	KH201010	STU22726	STU22816	STA22820	STH22820
Number ofMiseq reads	2,046,529	1,607,926	1,596,029	2,761,199	3,300,471	3,402,395	3,258,849
Number of MinION reads (N50 value (bp))	31,851 (17,748)	36,090 (18,178)	62,557 (16,480)	27,805 (17,032)	28,215 (18,035)	35,706 (20,059)	10,628 (23,095)
Number of circular contigs	3	3	3	3	3	3	3
Total length (bp)	3,752,265	3,783,477	3,784,219	3,789,154	3,787,795	3,781,142	3,781,292
Chromosome size (bp)	3,738,664	3,771,942	3,771,945	3,771,966	3,771,947	3,765,283	3,765,444
Plasmid sizes (bp)	3,960 and 6,512	3,961 and 6,512	3,961 and 6,512	3,961 and 6,512	3,950 and 6,512	3,961 and 6,512	3,950 and 6,512
GC content (%)	57.4	57.4	57.4	57.4	57.4	57.4	57.4
Coverage (×)	143	136	177	229	276	274	212
N50 value (bp)	3,738,664	3,771,942	3,771,945	3,771,966	3,771,947	3,765,283	3,765,444
Number of coding sequences	3,466	3,477	3,457	3,479	3,479	3,472	3,499
Number of rRNAs	25	25	25	25	25	22	22
Number of tRNAs	97	97	97	97	97	96	96
ST types	26	26	26	26	26	26	26
Whole-genome sequence accession no.	AP028097 for the chromosome, and AP028098 and AP028099 for the plasmids	AP028102 for the chromosome, and AP028103 and AP028104for the plasmids	AP028106 for the chromosome, and AP028107 and AP028108for the plasmids	AP028110 for the chromosome, and AP028111and AP028113for the plasmids	AP028115 for the chromosome, and AP028116and AP028118for the plasmids	AP028119 for the chromosome, and AP028120and AP028122for the plasmids	AP028123 for the chromosome, and AP028124and AP028126for the plasmids
DRA accession no.	DRX528540 for MiSeq data and DRX528541 for MinION data	DRX473524 for MiSeq data andDRX473525for MinION data	DRX473526 for MiSeq data andDRX473527for MinION data	DRX473512 for MiSeq data andDRX473513for MinION data	DRX473514 for MiSeq data andDRX473515f or MinION data	DRX473516 for MiSeq data andDRX473517 for MinION data	DRX473518 for MiSeq data andDRX473519 for MinION data

## Data Availability

This whole-genome shotgun project has been deposited at DDBJ/EMBL/GenBank. The versions presented here are the initial versions. Read archives have been deposited in DDBJ DRA. Genome nucleotide and DRA accession numbers are provided in Table 1.
